# Genetic and Neurological Deficiencies in the Visual System of *mct8* Mutant Zebrafish

**DOI:** 10.3390/ijms23052464

**Published:** 2022-02-23

**Authors:** Rotem Rozenblat, Adi Tovin, David Zada, Ilana Lebenthal-Loinger, Tali Lerer-Goldshtein, Lior Appelbaum

**Affiliations:** 1 The Faculty of Life Sciences, Bar-Ilan University, Ramat-Gan 5290002, Israel; rotemroze@gmail.com (R.R.); aditovin@gmail.com (A.T.); david.zada85@gmail.com (D.Z.); ilanalebenthal@gmail.com (I.L.-L.); tali.lerer@gmail.com (T.L.-G.); 2 The Multidisciplinary Brain Research Center, Bar-Ilan University, Ramat-Gan 5290002, Israel

**Keywords:** thyroid hormones, monocarboxylate transporter 8, opsin, zebrafish, OKR, pretectum

## Abstract

Thyroid hormones (THs; T3 and T4) enter cells using specific transporters and regulate development and metabolism. Mutation in the TH transporter monocarboxylate transporter 8 (MCT8, SLC16A2) is associated with brain hypothyroidism and neurological impairment. We established *mct8* mutant (*mct8*−/−) zebrafish as a model for MCT8 deficiency, which causes endocrinological, neurological, and behavioral alterations. Here, we profiled the transcriptome of *mct8*−/− larvae. Among hundreds of differentially expressed genes, the expression of a cluster of vision-related genes was distinct. Specifically, the expression of the opsin 1 medium wave sensitive 2 (*opn1mw2*) decreased in two *mct8* mutants: *mct8*−/− and *mct8^−25bp^*−/− larvae, and under pharmacological inhibition of TH production. Optokinetic reflex (OKR) assays showed a reduction in the number of conjugated eye movements, and live imaging of genetically encoded Ca^2+^ indicator revealed altered neuronal activity in the pretectum area of *mct8^−25bp^*−/− larvae. These results imply that MCT8 and THs regulate the development of the visual system and suggest a mechanism to the deficiencies observed in the visual system of MCT8-deficiency patients.

## 1. Introduction

Insufficient production of the thyroid hormones (THs), triiodothyronine (T3) and thyroxine (T4), and altered TH transport into the cells during prenatal and postnatal periods causes profound neurodevelopmental disorders [1,2]. In the nervous system, THs primarily regulate terminal neuronal differentiation processes, such as dendritic and axonal growth, synaptogenesis, cell migration, and myelination [3,4]. Several transmembrane TH transporters have been functionally described, including the monocarboxylate transporter 8 (MCT8) [5,6]. The critical role of MCT8 in regulating TH signaling, metabolism, and brain development is evident by the symptoms of the X-linked inherited Allan–Herndon–Dudley syndrome (AHDS/MCT8-deficiency) associated with mutations in the MCT8 (SLC16A2) gene. MCT8-deficiency is characterized by severe cognitive deficits, spastic quadriplegia, hypotonia, and elevated serum T3 levels [7,8]. Notably, abnormal conjugating eye movements and strabismus were also diagnosed in the patients [6,8,9], suggesting an altered visuomotor function in MCT8-deficient patients. 

The visual system includes retinal photoreceptors, which are specialized cells that house light-sensitive visual pigments called opsins. Opsins are integral membrane proteins covalently linked to a light-sensitive vitamin A-based chromophore [10]. The opsin family is highly conserved across phylogeny and isolated in animals, ranging from cnidarians to primates [11]. In rodents, THs regulate the expression and differentiation of opsins in the visual system [12,13]. Similarly, in human organoids, THs regulate photoreceptor subtype specification [14]. These findings suggest that abnormal cellular transport of THs can affect the expression and function of opsins and the development of the visual system in MCT8 deficiency.

To study the mechanism of MCT8 deficiency, several mouse models have been generated and extensively studied [15,16,17,18,19,20]. A complementary animal model is the zebrafish, which is a simple vertebrate with a conserved organization of the central nervous system (CNS) and the hypothalamus–pituitary–thyroid gland axis [21,22]. Similar to mammals, THs are essential for the development and function of zebrafish, and the lack of endogenous and maternal TH impairs neuronal development in embryos [23]. We generated *mct8* mutant (*mct8*−/−) zebrafish. The transparent nature of the *mct8*−/− larvae enabled revealing a wide range of neurological and behavioral deficits, including hypomyelination, reduced axonal branching and synaptic density, altered locomotor activity, and sleep [24,25,26]. However, the role of Mct8 in regulating the function of the visual system is unclear. Here, we performed whole transcriptome sequencing, TH-related pharmacological assays, and time-lapse imaging of neuronal activity and behavior to characterize genetic and neuronal alterations in *mct8*−/− larvae, with special emphasis on the visual system.

## 2. Results

### 2.1. Profiling the Transcriptome of mct8−/− Larvae Revealed Altered Expression of Visual System Genes

**Figure 1:**
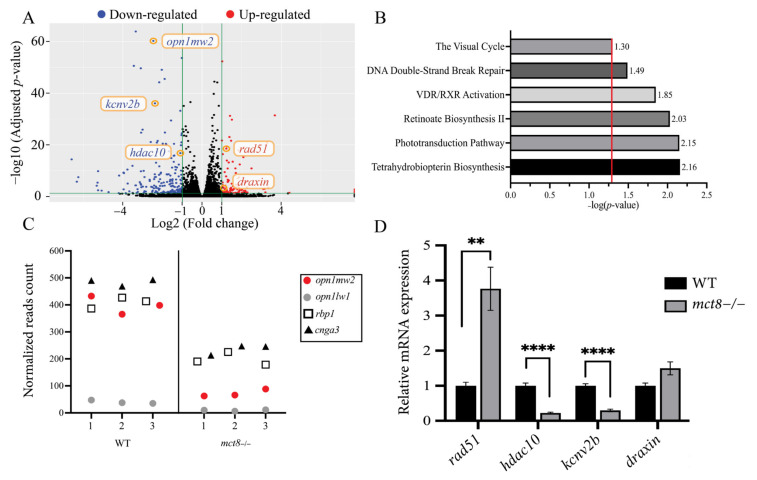
Transcriptome profiling in *mct8*−/− larvae. (**A**) Differentially expressed genes in 6 days post-fertilization (dpf) *mct8*−/− and wild-type (WT) sibling larvae (*n* = 3 batches for each genotype). Green vertical lines differentiate between significant and insignificant expressed genes. Blue and red dots mark down-regulated and up-regulated genes (1 < log2 [fold change] < 1, adjusted *p*-value (*p*-adj) < 0.05), respectively. Black dots mark insignificant expressed genes. Orange circles mark genes validated by qRT-PCR. (**B**) Representative enriched gene pathways in *mct8*−/− larvae. The red vertical line marks the threshold of significantly enriched pathways (*p*-adj ≤ 0.05). (**C**) Normalized reads count of four differentially expressed genes selected from the enriched vision pathways shown in B. X-axis represents the sample number in each genotype. (**D**) Relative gene expression of selected genes was quantified using qRT-PCR and normalized against *actb* (*n* = 5 batches of larvae for each group). Statistical significance determined by *t*-test: two-sample. ** *p* < 0.01, **** *p* < 0.0001.

Loss of function of MCT8 affects TH transport and the expression of numerous genes in vertebrates [27,28,29]. To profile the transcriptome of the zebrafish model for MCT8 deficiency, we performed RNA-seq in 6 days post-fertilization (dpf) *mct8*−/− larvae and their wild-type (WT) siblings. Differentially expressed genes were determined using the DESeq R Bioconductor package, with a criterion of −1 < log2 (fold change) < 1 and adjusted *p*-value (*p*-adj) < 0.05. The analysis identified 97 and 204 up-regulated (Table S1) and down-regulated (Table S2) genes, respectively, in *mct8*−/− larvae (Figure 1A). To identify and classify specific genetic pathways, we utilized the Ingenuity Pathway Analysis (IPA) software and found 23 significantly enriched canonical pathways (*p*-adj ≤ 0.05). Among these pathways, prominent alterations were found in visual-system-related pathways, such as the visual cycle, retinoate biosynthesis II and phototransduction (Figure 1B). In addition, marked enrichment was found in the VDR/RXR activation pathway, which includes vitamin D receptor-related genes, and is related to hypothyroid diseases [30]. Additional enriched processes include the DNA double-strand break repair and the tetrahydrobiopterin biosynthesis pathways [31]. Taking into account the prominent alteration in the visual system pathways, we profiled the expression of some of the visual genes that are related to these pathways (Figure 1C). The expression of the long-wave-sensitive 1 opsin1 (*opn1lw1*) and medium-wave-sensitive 2 opsin 1 (*opn1mw2*) were reduced by 4.35-fold (*p*-adj = 1.89 × 10^−7^) and 5.52-fold (*p*-adj = 3.72 × 10^−65^), respectively. The expression of the retinol-binding protein 1 (*rbp1*) and cone photoreceptor cyclic nucleotide-gated channel alpha 3 (*cnga3*) was reduced by 2.06-fold (*p*-adj = 9.78 × 10^−22^) and 2.05-fold (*p*-adj = 1.6 × 10^−24^), respectively (Figure 1C). 

Next, we validated the transcriptome results in selected genes of interest using quantitative real-time PCR (qRT-PCR) on cDNA produced from separate batches of 6 dpf *mct8*−/− larvae and their WT siblings. The expression of the DNA repair gene *rad51* and the structural chromatin modulator *hdac10* was up-regulated by 3.76-fold (*p* = 0.002) and down-regulated by 4.54-fold (*p* = 1.36 × 10^−5^), respectively. In addition, the expression of the voltage-gated potassium channel *kcnv2b* was reduced by 3.45-fold (*p* = 8.52 × 10^−8^), while the expression of the axon guidance regulator *draxin* did not change (Figure 1D). Taken together, these results identified dozens of differentially expressed genes in various pathways, including the visual system in *mct8*−/− larvae.

### 2.2. The Expression of opn1mw2 Is Reduced in mct8−/− Larvae and Is Regulated by THs

**Figure 2:**
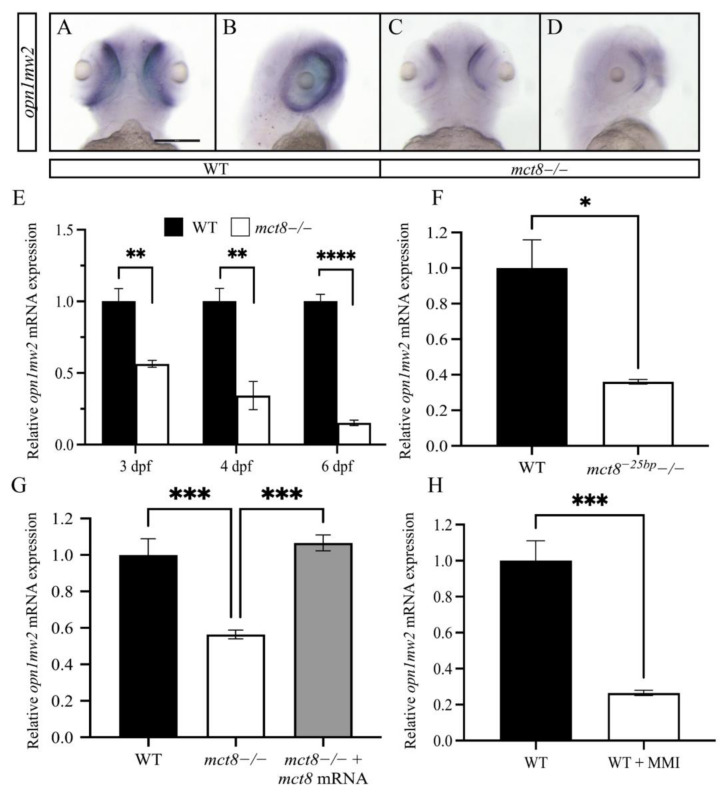
The expression of *opn1mw2* is reduced in *mct8*-deficient larvae. (**A**–**D**) Ventral (**A**,**C**) and lateral (**B**,**D**) views of the spatial expression pattern of *opn1mw2* mRNA in 3 dpf WT (**A**,**B**) and *mct8 *−/− (**C**,**D**) larvae. Scale bar = 300 μm. (**B**–**E**) The relative levels of *opn1mw2* mRNA were determined using qRT-PCR. (**E**) Expression levels in 3, 4, and 6 dpf *mct8*−/− and WT sibling larvae (*n* = 5 batches of larvae for each group). (**F**) Expression levels in 6 dpf *mct8^−25bp^*−/− larvae (*n* = 5 batches of larvae for each group). (**G**) Expression levels in 3 dpf *mct8*−/−, WT siblings, and *mct8* mRNA-injected *mct8*−/− embryos (*n* = 5 batches of larvae for each group). (**H**) The expression levels of *opn1mw2* in WT larvae treated with the TH inhibitor methimazole (MMI) (*n* = 5 batches of larvae for each group). Relative gene expression was normalized against *actb* in all assays. Statistical significance determined by *t*-test: two-sample. * *p* < 0.05, ** *p* < 0.01, *** *p* < 0.001, **** *p* < 0.0001.

The transcriptome data showed robust reduction in the expression of the vision-related gene *opn1mw2*. In order to study the effect of MCT8-deficiency on the expression of *opn1mw2* during zebrafish development, we monitored the spatial expression pattern of *opn1mw2*, using in situ hybridization (ISH), in WT and *mct8*−/− larvae. At 3 dpf, the expression of *opn1mw2* reduced in the forebrain and predominantly in the retina of *mct8*−/− larvae (Figure 2A–D). To study the dynamic expression of *opn1mw2* along development, qRT-PCR assays were performed. The levels of *opn1mw2* mRNA reduced by 1.78-, 2.94-, and 6.66-fold in 3, 4, and 6 dpf *mct8*−/− larvae, respectively (Figure 2E). To verify the specificity of this phenotype to *mct8*, we reevaluated these results in additional *mct8* mutant (*mct8^−25bp^−/−)* larvae. This mutant, which harbors a 25 bp deletion in exon 3, demonstrated a 2.77-fold reduction in the expression of *opn1mw2* in 6 dpf larvae (Figure 2F), as was the case in *mct8*−/− larvae. Furthermore, microinjection of *mct8* mRNA at one-cell stage rescued and normalized the expression of *opn1mw2* in 3 dpf *mct8*−/− embryos (Figure 2G). These results show that Mct8 mediates *opn1mw2* expression. 

Considering that lack of Mct8 can affect TH transport, we tested whether THs regulate *opn1mw2* expression. Larvae were treated with 6 nM of the TH inhibitor methimazole (MMI) [32] over 4 consecutive days. Similar to the findings in *mct8*−/− larvae (Figure 2E–G), the expression of *opn1mw2* mRNA decreased by 3.84-fold (Figure 2H) in MMI-treated 6 dpf larvae, suggesting that THs can induce the expression of *opn1mw2* and regulate the development of the visual system.

### 2.3. The Number of Conjugated Eye Movements Is Reduced in mct8^−25bp^−/− Larvae

**Figure 3:**
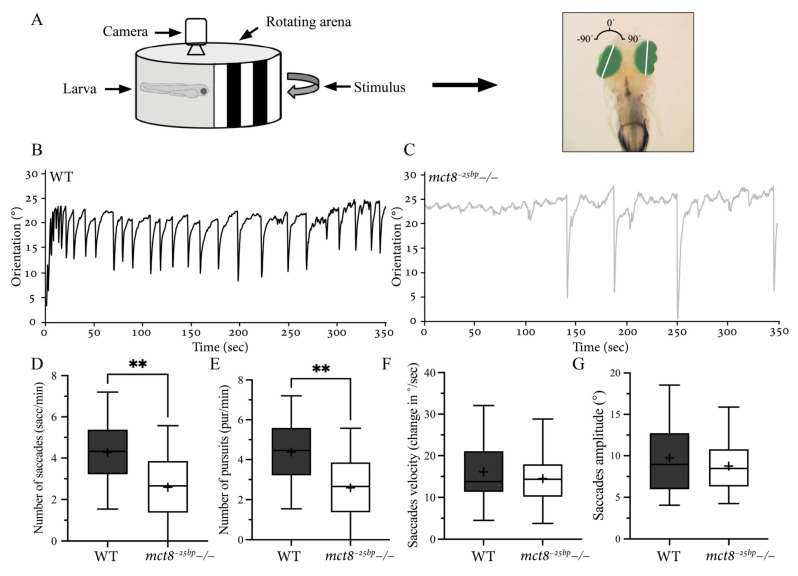
Altered visuomotor behavior in *mct8^−25bp^*−/− larvae. (**A**) Experimental optokinetic reflex (OKR) setup. Video tracking of the eyes under the stimulus of moving vertical black-and-white stripes. The eyes (green region) were distinguished from the background, and the angular orientation of the long axis of each eye (white line) was analyzed. Clockwise and counterclockwise eye orientation was defined as positive and negative angles, respectively. (**B**,**C**) The representative curve of the average dynamic orientation of both eyes in 6 dpf WT (**B**) and *mct8^−25bp^*−/− (**C**) larvae. (**D**–**G**) Box plots demonstrate the number, amplitude, and velocity of saccades, as well as the number of pursuits in *mct8^−25bp^*−/− (*n* = 17) and WT sibling (*n* = 18) larvae. The ‘+’ sign shows the mean. Statistical significance was determined by Mann–Whitney U test. ** (*p* < 0.01).

The altered expression of many visual genes (Figure 1), such as the retinal *opn1mw2* (Figure 2) in *mct8*−/− larvae, suggests that loss of Mct8 results in neuronal and behavioral deficiencies of the visual system. Thus, we studied visuomotor behavior in *mct8^−25bp^*−/− larvae. A well-established assay in zebrafish larvae is the optokinetic reflex (OKR), which is induced as a response to a motion stimulus [33,34]. In all vertebrates, an OKR is a combination of slow eye movements following a moving stimulus (pursuits) and fast eye movements in the opposite direction to the stimulus (saccades) in both eyes simultaneously. Both phases are important for optimal visual acuity, orientation, and prey-capture behavior [35]. To monitor OKR in *mct8^−25bp^*−/− and their WT siblings, larvae were mounted in 3% methylcellulose, and their eyes were stimulated using rotating vertical black-and-white stripes (Figure 3A). The amplitude and duration of the saccades and pursuits in both eyes were quantified (Figure 3B,C). Notably, the number of saccades and pursuits reduced in *mct8^−25bp^*−/− larvae (Figure 3D,E), while the amplitude and velocity of the saccades did not change (Figure 3F,G). Analyzing the same parameters, no differences were found between the left and right eyes. These results show that although the pattern of the saccades and pursuits seems normal, the rate of conjugated eye movement is reduced in *mct8^−25bp^*−/− larvae. 

### 2.4. Altered Spontaneous Neuronal Activity in the Pretectum of mct8^−25bp^−/− Larvae

**Figure 4:**
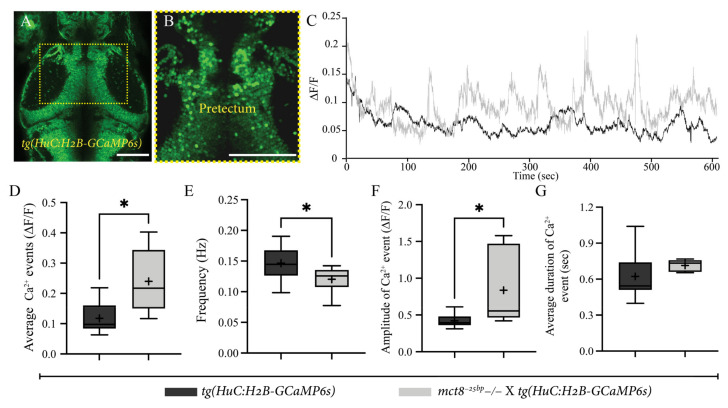
Stronger and slower neuronal activity in the pretectum area in *mct8*^−*25bp*^−/− larvae. (**A**) Dorsal view of *tg(HuC:H2B-GCaMP6s)* 6 dpf larva (head pointing to the top). The dashed yellow square marks the enlarged view of the pretectum shown in (**B**). Scale bar = 50 µm. (**C**) Average relative fluorescence variation (∆F/F) activity of [*mct8*^−*25bp*^−/− × *tg(HuC:H2B-GCaMP6s)*] (grey) and their *tg(HuC:H2B-GCaMP6s)*-siblings (black). (**D**–**G**) Changes in single-cell Ca^2+^ events are presented by the average number of events (**D**), frequency (**E**), amplitude (**F**) and duration (**G**). The ‘+’ sign shows the mean. *tg(HuC:H2B-GCaMP6s)*-siblings: *n* = 9, [*mct8*^−*25bp*^−/− × *tg(HuC:H2B-GCaMP6s)*]: *n* = 6). Statistical significance was determined by Mann–Whitney U test. * (*p* < 0.05).

The abnormal conjugated eye movement found in *mct8^−25bp^*−/− larvae elicited us to study the activity of neurons in the pretectum area. In vertebrates, the pretectum regulates visual neuronal pathways such as the OKR [36,37]. Two-photon live imaging of the genetically encoded calcium indicator GCaMP6s was used to monitor neuronal activity in the pretectum of 6 dpf [*mct8*^−*25bp*^−/− × *tg(HuC:H2B-GCaMP6s)*] and their *tg(HuC:H2B-GCaMP6s*) siblings (Figure 4A–C). Calculation of the average relative fluorescence variation (∆F/F) per single neuron showed increased activity in [*mct8*^−*25bp*^−/− × *tg(HuC:H2B-GCaMP6s)*] larvae (Figure 4D). This result coincides with a decrease in the frequency of Ca^2+^ events and the increase in average maximum amplitude in single neurons of [*mct8*^−*25bp*^−/− × *tg(HuC:H2B-GCaMP6s)*] larvae (Figure 4E,F, respectively). The average duration of each Ca^2+^ event did not change (Figure 4G). Altogether, these results show that the activity of pretectal neurons is higher and slower in *mct8*^−*25bp*^−/− larvae. These deficiencies in pretectal activity are associated with reduced OKR and altered expression of genes regulating the visual system, including the opsin gene *opn1mw2*, in Mct8-deficient zebrafish. 

## 3. Discussion

THs are vital to the function of various essential processes, including cell growth and differentiation, as well as the development of the nervous system [1]. Insufficient transport of THs into the cells due to a mutation in the MCT8 transporter causes severe intellectual and motoric disability. We performed whole transcriptome sequencing, live imaging of neuronal activity, and behavioral experiments in a zebrafish model for MCT8-deficiency. The results identified differentially expressed genes, including a reduction in the mRNA levels of *opn1mw2* and decreased conjugated eye movement. In addition, imaging of spontaneous neuronal activity in the pretectum revealed slower and stronger Ca^2+^ events in Mct8-deficient larvae. The results suggest genetic and neurological mechanisms that can regulate the abnormal function of the visual system in Mct8-deficiency.

The transcriptome profiling of *mct8*−/− larvae revealed dozens of differentially expressed genes. Primarily, the expression of vision-related genes was altered, including reduced *opn1mw2* mRNA levels, which are expressed in the retina [38]. This reduced expression is likely due to insufficient levels of THs in the cells, because pharmacological inhibition of TH synthesis resulted in reduced *opn1mw2* expression in WT zebrafish larvae. This interaction between the thyroid system, opsins, and the visual system was observed in additional vertebrates. For example, TH controls adult cone opsin expression in mice [39]. TH receptors are expressed in the retina of early-stage chick embryos [40], and impaired cells were found in the retina of a hypothyroid rat [41]. In addition, supporting our findings in *mct8* mutant zebrafish, alteration in retinal precursor cells affected the cone function in MCT8-knockdown chickens [42]. Transcriptome analysis in *mct8* Morpholino-injected 25 h post-fertilization (hpf) embryos revealed hundreds of differentially expressed genes. Among them, the expression of the vision-related gene *rorab* was reduced in *mct8* Morpholino-injected embryos, suggesting that maternal TH regulates the expression of *rorab* and the development of the eyes [28]. The reduced expression of *rorab* may also be associated with the neurological deficiencies observed in *mct8*−/− 6 dpf larvae. The results of this work link TH function with *opn1mw2* expression in Mct8-deficient zebrafish and could be tested also in mammals. 

The effect of Mct8 on visual gene expression, specifically the reduced expression of *opn1mw2*, suggests that the function of the eyes and visual brain nuclei is deficient. The OKR is a hallmark visual process in all vertebrates ranging from fish to humans [43]. In *mct8^−25bp^*−/− larvae, we found a reduced number of saccades and pursuits. Similarly, a mutation in *mfrp*, a gene associated with retinal degeneration, reduced the number of saccades in zebrafish mutant [44]. These results suggest that reduced expression of *opn1mw2* combined with altered expression of array of visual genes can cause abnormal OKR behavior.

The OKR deficiencies could reflect an abnormal function of the visual pathway from the eye to the brain. Optic flow directions are processed by direction-sensitive neurons, which are abundant in the zebrafish pretectum [45]. Similarly, the activity of pretectal neurons is associated with the onset of eye movements, including saccades and pursuits, in mammals [46]. Zebrafish pretectal activation induced conjugated eye movements while inhibition resulted in a lack of response, suggesting that the pretectum regulates the OKR movement [47]. In *mct8^−25bp^*−/− larvae, the amplitude of Ca^2+^ events increased, while the frequency decreased in the pretectum. Taking into account that most neurons in the pretectum are tuned to binocular directional movements [48,49], lack of Mct8 may alter their development and function, resulting in dysregulated conjugating eye movements.

The abnormalities in the visual system of MCT8-deficiency patients have not been thoroughly studied. This study associates changes in visual gene expression with altered neuronal activity in the pretectum and abnormal OKR behavior in zebrafish model for MCT8-deficiency. Future functional research of the specific genes using gain- and loss-of-function techniques, which are well established in zebrafish [26,50], combined with simultaneous monitoring of eye movement and neuronal activity, is expected to elucidate the underlined mechanism of MCT8-deficiency. In addition, live imaging of the neuronal response to visual stimulus in the entire brain [51,52,53] of the transparent *mct8^−25bp^*−/− larvae could provide important insights to understand the effect of Mct8 on the activity of compatible neuronal circuits. Future experiments in the *mct8^−25bp^*−/− zebrafish model, which will combine functional assays with pharmacological and genetic treatments [25,54,55,56,57,58] could lead to the development of a therapeutic strategy to MCT8-deficiency and other TH resistance disorders. 

## 4. Materials & Methods

### 4.1. Zebrafish Husbandry, Mutant, and Transgenic Lines

Adult zebrafish were raised and maintained in fully automated zebrafish housing systems (Aquazone, Zofit, Israel; temperature 28 ± 0.5 °C, pH 7.0, conductivity 500 μS) under 14 h light/10 h dark cycles and fed twice a day. Embryos were produced by natural spawning and raised in egg-water containing methylene blue (0.3 ppm) in a light-controlled incubator at 28 ± 0.5 °C, as previously described [59]. 

In this study, we used the previously established *mct8*−/− line [23] and the *tg(HuC:H2B-GCaMP6s)* line (also named *tg(elavl3:H2B-GCaMP6s)*) [60]. All animal protocols were reviewed and approved by the Bar-Ilan University Bioethics Committee.

The CRISPR system [61] was used to establish the *mct8^−25bp^*−/− line. The Cas9 mRNA was transcribed using the Cas9 expression vector (MLM3613, Addgene #42251, Watertown, MA, USA) digested with PmeI and the mMESSAGE mMACHINE T7 ULTRA Kit (Life Technologies, Grand Island, NY, USA), and poly-A tailing was added using Poly(A) Tailing Kit (Ambion^®^, Life Technologies, Grand Island, NY, USA). Annealed oligonucleotides (5′-TAGGTGCAGGCGCTGCTGGCCC-3′ and 5′-AAACGGGCCAGCAGCGCCTGCA-3′), designed by the ZiFiT Targeter website (http://zifit.partners.org/, accessed on 9 March 2019) to target the specific genomic site (5′-GGTGCAGGCGCTGCTGGCCC-3′) in the third exon of the *mct8* gene, were cloned into BsaI-digested gRNA expression vector (pDR274, Addgene #42250, Watertown, MA, USA). sgRNA were transcribed using the DraI-digested gRNA expression vectors as templates and the RNA synthesis protocol (#M0255, New England BioLabs Inc., Ipswich, MA, USA). Approximately 300 ng/µL of Cas9 mRNA and 15 ng/µL of sgRNA were co-injected to one-cell stage WT embryos. These mosaic embryos were raised to adulthood and out-crossed with WT fish to identify F0 founder fish. F1 heterozygous fish, which carry a 25 bp deletion mutation in the targeted site, were identified by PCR using the following primers: 5′-GCTGCGGCTCCTCGTTTGCATT-3′ and 5′-GCACAGCAGTGGCGACGCCAAAG-3′, and sequencing and selected F1 individuals were out-crossed with WT fish. The F2 heterozygous progeny were inter-crossed to generate the homozygous *mct8^−25bp^*−/− line. 

### 4.2. Whole Transcriptome RNA-seq and Pathway Analysis

Total RNA was extracted from three groups of 6 dpf WT and *mct8*−/− zebrafish (30 larvae per sample in a total of 6 samples), using the Direct-zol RNA MiniPrep Kit (Zymo Research Corporation) according to the manufacturer’s protocol. The quality and quantity of each RNA sample were assessed by Agilent’s 2100 Bioanalyzer 6000 Pico Kit (Agilent Technologies, Santa Clara, CA, USA). One µg of total RNA was used as input for preparations of RNA libraries using TrueSeq RNA Library Prep Kit v2 (Illumina, San Diego, CA, USA) according to the manufacturer’s protocol. Six libraries were loaded on one lane of an Illumina HiSeq2500 instrument 100 bp paired-end run at the Technion Genome Center. Pair-end RNA-seq protocol was used, yielding about 21–35 million paired reads per sample. Paired-end reads were aligned to the zebrafish genome (Zv9) using the STAR RNA-seq aligner software (version STAR_2.3.0e) [62]. The STAR genome database was built with the ENSEMBL annotation file (release 77, Danio_rerio.Zv9.77.gtf). Only uniquely mapped genes were considered for further analysis. Raw read counts for 33737 Ensembl-annotated gene-level features were determined using HTSeq-count [63]. Differentially expressed genes were determined with the R Bioconductor package DESeq [64]. *p*-Values were corrected with Benjamini–Hochberg FDR procedure. Genes with adjusted *p*-values < 0.05 and |log fold change| > 1 were considered as differentially expressed.

This list was then submitted to the IPA software (www.ingenuity.com, accessed on 12 October 2021). The list is mapped by Ingenuity based on ortholog information for humans, mouse, and rat. Ingenuity uses public databases to determine significantly enriched canonical pathways from the gene list. A total of 56 significantly changed genes were mapped and included in the analysis. The significance of the pathways was determined from a *p*-value < 0.05 of overlap, calculated using a right-tailed Fisher’s exact test.

### 4.3. Quantitative Real-Time PCR

The relative mRNA levels of *opn1mw2* (Ensembl number: ENSDARG00000044280), *hdac10 (*ENSDARG00000086458*), rad51* (ENSDARG00000041411)*, kcnv2b* (ENSDARG00000062906), *draxin* (ENSDARG00000058256) and *actin* (NCBI accession number: AY222742.1) were determined using qRT-PCR. Total mRNA was extracted from five groups of *mct8+/+* and *mct8*−/− 6 dpf larvae (*n* = 5 batches per group, *n* = 8–10 larvae per batch), using Direct-zol RNA MiniPrep kit (Zymo Research Corporation) according to the manufacturer’s instructions. One μg mRNA was reverse-transcribed using qScript cDNA SuperMix (Quanta BioSciences, Gaithersburg, MD, USA). Relative transcript levels were determined by the 7900HT Fast Real-Time PCR System (Applied Biosystems, Foster City, CA, USA). Triplicates of each cDNA sample were PCR-amplified using the PerfeCTa SYBR Green FastMix (Quanta BioSciences, Gaithersburg, MD, USA) and the following specific primers: *opn1mw2*: 5′-TGGCAATTAATTCTACGCAG-3′ and 5′-TTGCTGGTGTCTTCTTGGCA-3′ *hdac10*: 5′-TCTGACTCTGGGAGATTGGG-3′ and 5′-GGATTTTGATTGTGGACTGGG-3′ *kcnv2b*: 5′-CGAAGGAAATGCAGCCAAAG-3′ and 5′-CGTCCCTAAAACCAACTTTACC-3′ *draxin:* 5′-CCGTGGCCTAAATAACAAGTG-3′ and 5′-CTTGGTTGCTGTTGACTGATTC-3′ *rad51*: 5′-ATGGTCAGTGTTGAAGGCTC-3′ and 5′-GGGTGGAGGTGAAGGAAAAG-3′. The relative quantification of each gene expression was normalized against the housekeeping gene *actb*: 5′-CAACAGGGAAAAGATGACACAG-3′ and 5′-CATCACCAGAGTCCATCACG-3′ and subjected to the ΔΔ*C*T method [65].

### 4.4. Whole-Mount In Situ Hybridization (ISH) and Probe Preparation

To prepare mRNA antisense probes, the full coding sequence of *opn1mw2* was amplified. The PCR products were cloned into a pCRII-TOPO (Invitrogen, Carlsbad, CA, USA), which served as a template to transcribe digoxigenin-labeled antisense mRNA probes for *opn1mw2*. At 3 dpf, WT sibling larvae were fixed in 4% paraformaldehyde overnight at 4 °C and stored in 100% methanol. The location and level of mRNA expression were detected by whole-mount ISH, as described previously [66]. Each sample was incubated overnight with DIG-AP antibody (Roche, Basel, Switzerland), diluted to 1:2500, and then stained with BM purple (Roche, Basel, Switzerland). The reaction was terminated after being washed 3 times with PBT. 

### 4.5. MMI Pharmacological and Transient Expression Assays

Stock solution of 250 mM MMI in 12.5% ethanol was used for TH inhibition assays in 52 hpf embryos, which were placed in 25 ml water containing final concentration of 6 nM MMI (CAS-RN 60-56-0, purity ≥99%, Sigma–Aldrich, St. Louis, MO, USA) or 0.07% ethanol as a control. At 6 dpf, larvae were sampled and subjected to quantification of *opn1mw2* expression. In rescue experiments, 2 µL of in vitro transcribed *mct8* mRNA (100 ng/µL) was microinjected into *mct8*−/− embryos at the one-cell stage. Preparation of *mct8* mRNA was performed as previously described [23].

### 4.6. Imaging and Image Analysis

Larvae stained with RNA probes were imaged using an epifluorescent stereomicroscope (Leica M165FC, Wetzlar, Germany). All images were taken using Leica Application Suite imaging software V3.7 (Leica, Wetzlar, Germany). 

Imaging of neuronal activity in the pretectal area was conducted on 6 dpf larvae, mounted with 1.5% low-melting-point agarose. Imaging was performed using a Zeiss LSM710 upright confocal/two-photon microscope (Zeiss, Oberkochen, Germany) with ×20, 1.0 NA objective and the Mai-Tai 2-photon laser (Spectra-Physics, Santa Clara, CA, USA), tuned to 920 nm excitation wavelength. Half-hour movies (approximately 7000 frames) were taken at a speed of ~4.12 frames per second (fps), using an image resolution of 256 × 256 pixels (~0.223-mm^2^ optical plane). All images were processed using ImageJ (National Institutes of Health, Bethesda, MD, USA). 

### 4.7. OKR Assay

OKR experiments were carried out using a custom-made device. The device is composed of a portable rotating drum placed above a light source. The interior of the drum contains black-and-white vertical stripes (~0.7 cm in width) as a stimulus. The drum rotates using an electrical motor. The 6 dpf larvae were fixed in 3% methylcellulose, and placed in a 5.5 cm petri dish inside the drum, while the stripes were turning clockwise. The eye movements of the larvae were recorded for 350 s in 2.85 Hz, with an epifluorescent stereomicroscope (Leica M165FC, Wetzlar, Germany) equipped with an AmScope 10 MP C-Mount Microscope Camera MU1000, and then analyzed using the AmScope v4.8.1 software (AmScope, Irvine, CA, USA). Experiments were conducted blindly to fish genotype, and each larva was genotyped following the video recording. Eye movement properties were detected using an open-source MATLAB program [67]. This data were then analyzed in a custom MATLAB software based on a previously published script [51], and saccades and pursuits were defined based on a threshold of amplitude and velocity. 

### 4.8. Genotyping

Genotyping of *mct8^−25bp^*−/− zebrafish was conducted as follows. Genomic DNA was extracted from larvae, then incubated in 100 µL of 50 nM NaOH at 95 °C for 20 min, and then kept on ice. Next, 10 µL of Tris (pH = 8) was added, and the sample was subjected to 30-s vortex treatment and 2 min spinning at 12,000 relative centrifugal force (RCF). The extracted genomic DNA was then amplified by PCR using the following primers: 5′-AAACAGCACAGCAGTGGCGACG-3′ and 5′-GACTCTGGGTCTCTGGTACTTCAC-3′. PCR product was then run on 3% agarose gel for 90 min. PCR amplification resulted in fragments of 454 bp in WT and 429 bp in *mct8^−25bp^*−/− zebrafish. Selected PCR products were sequenced to validate the gel results. As expected, heterozygous fish showed two DNA fragments, indicating the presence of both mutated and WT *mct8* alleles.

### 4.9. Data and Statistical Analysis

Determination of sex in larval zebrafish before 8 dpf cannot be identified [68], therefore larvae used in all experiments are of unknown sex. Bar graphs, box plots, as well as statistical analysis were generated using GraphPad Prism version 9.2.0 for macOS (GraphPad Software, San Diego, CA, USA). Quantification of ΔF/F in pretectum neurons was determined and produced using published MATLAB scripts [69]. Ca^2+^ events were determined by applying a threshold equal to two times the standard deviation of the baseline fluctuations in each cell. Analysis was applied on all fluctuations exceeding this threshold.

## Supplementary Materials

The following supporting information can be downloaded at: www.mdpi.com/article/10.3390/ijms23052464/s1.
