# Supplementary material for: Genetic and Neurological Deficiencies in the Visual System of mct8 Mutant Zebrafish
Source: Int J Mol Sci. 2022 Feb 23;23(5):2464. doi: 10.3390/ijms23052464 (PMC8910067; doi:10.3390/ijms23052464)
Supplement: Supplementary file 1 [file ijms-23-02464-s001.zip › ijms-1594064-supplementary.pdf]

Supplementary Material

Table S1. Differential expression of up-regulated genes.

	Transcript ID	Transcript Name	Fold Change
1	ENSDARG00000081136	*dre-mir-132-3*	Inf
2	ENSDARG00000079937	*pcdh2g5*	Inf
3	ENSDARG00000080818	Uncharacterized protein	Inf
4	ENSDARG00000076704	MRTFB	21.23892955
5	ENSDARG00000026403	*hephl1b*	12.72172318
6	ENSDARG00000093515	*sfxn5a*	10.07125596
7	ENSDARG00000069223	*pcdh1g26*	9.684602393
8	ENSDARG00000086493	*mtnr1bb*	9.152538927
9	ENSDARG00000098037	*si:ch73-65n21.1*	8.83954185
10	ENSDARG00000095720	*si:dkey-65b12.13*	8.532970048
11	ENSDARG00000075856	Uncharacterized protein	5.637323319
12	ENSDARG00000075080	Uncharacterized protein	4.892359412
13	ENSDARG00000096474	*si:dkey-16b10.3*	4.881154529
14	ENSDARG00000075785	*herc5.1*	4.843396558
15	ENSDARG00000014624	*wu:fj40e01*	4.771862089
16	ENSDARG00000076069	*ubald1a*	4.635896203
17	ENSDARG00000014657	*casp23*	4.529031804
18	ENSDARG00000092379	*si:dkeyp-51b9.3*	4.47033898
19	ENSDARG00000046057	Uncharacterized protein	4.376841696
20	ENSDARG00000090092	Uncharacterized protein	4.338706794
21	ENSDARG00000093054	*si:ch211-207c6.5*	4.326073939
22	ENSDARG00000014165	*ssr3*	4.226289286
23	ENSDARG00000004763	*hhla2a.2*	4.196435096
24	ENSDARG00000014309	*spaw*	4.072192944
25	ENSDARG00000093186	*si:dkey-30j10.5*	4.046362552
26	ENSDARG00000093541	Uncharacterized protein	3.988006752
27	ENSDARG00000088251	*si:ch211-24o10.6*	3.98273893
28	ENSDARG00000088733	*zmp:0000000938*	3.723668105
29	ENSDARG00000091918	*si:ch211-207c6.7*	3.723199294
30	ENSDARG00000088141	*sa30841*	3.652357422
31	ENSDARG00000086574	*si:dkey-146c18.1*	3.64809034

32	ENSDARG00000067964	*slc6a5*	3.646460582
33	ENSDARG00000070757	*si:ch211-155i14.1*	3.596154685
34	ENSDARG00000028396	*fkbp5*	3.557809869
35	ENSDARG00000040200	*si:dkeyp-86f5.1*	3.543809676
36	ENSDARG00000089570	*ccdc169*	3.476698594
37	ENSDARG00000032820	*rxfp2a*	3.452355035
38	ENSDARG00000088309	*eepd1*	3.427396754
39	ENSDARG00000078365	Uncharacterized protein	3.280674807
40	ENSDARG00000087380	*si:dkey-217g21.3*	3.253675079
41	ENSDARG00000058553	*sprb*	3.242806168
42	ENSDARG00000088022	*mhc1zfa*	3.234146896
43	ENSDARG00000074988	*si:ch211-209n20.1*	3.217235028
44	ENSDARG00000096431	*si:ch211-209j12.1*	3.196961749
45	ENSDARG00000052620	Uncharacterized protein	3.193918159
46	ENSDARG00000095126	*si:dkey-82i20.2*	3.074090214
47	ENSDARG00000035116	Uncharacterized protein	2.976497197
48	ENSDARG00000087287	*zgc:174180*	2.946110506
49	ENSDARG00000097318	*si:dkey-264c3.2*	2.941042614
50	ENSDARG00000041433	*si:dkey-7c18.24*	2.914216056
51	ENSDARG00000092191	*si:dkey-238c7.13*	2.856343134
52	ENSDARG00000097829	*si:ch211-152c8.5*	2.824343497
53	ENSDARG00000093260	*elovl4a*	2.821952589
54	ENSDARG00000070515	Uncharacterized protein	2.798088803
55	ENSDARG00000042667	*klf2a*	2.754758085
56	ENSDARG00000039501	*ugt2a6*	2.747446186
57	ENSDARG00000074748	*gvin1l*	2.69365121
58	ENSDARG00000026049	*mxf*	2.677170095
59	ENSDARG00000042396	*trim35-30*	2.659179714
60	ENSDARG00000007889	*csf1ra*	2.65272815
61	ENSDARG00000000796	*nr4a1*	2.594383597
62	ENSDARG00000088234	*si:ch211-217k17.12*	2.571749807
63	ENSDARG00000073709	*fam149a*	2.539747465
64	ENSDARG00000036767	*urgcp*	2.533444994
65	ENSDARG00000045015	*cyp27b1*	2.495123302
66	ENSDARG00000094702	*si:dkey-61n16.5*	2.474117277

67	ENSDARG00000056587	*cyp2r1*	2.449659608
68	ENSDARG00000070510	Uncharacterized protein	2.445839646
69	ENSDARG00000079429	Uncharacterized protein	2.440423856
70	ENSDARG00000040463	*slc25a51b*	2.387500662
71	ENSDARG00000090061	*si:dkeyp-86f5.2*	2.379231301
72	ENSDARG00000089934	*si:ch211-64i20.3*	2.378703885
73	ENSDARG00000059305	*tmem37*	2.372803851
74	ENSDARG00000051983	*dguok*	2.351599611
75	ENSDARG00000031001	*mettl25b*	2.333302324
76	ENSDARG00000041411	*rad51*	2.328150378
77	ENSDARG00000087122	Uncharacterized protein	2.316291089
78	ENSDARG00000043901	*ugt5g2*	2.277302298
79	ENSDARG00000071374	*lrrtm2*	2.2730158
80	ENSDARG00000055751	*fosb*	2.260763352
81	ENSDARG00000097198	*si:dkey-11n14.2*	2.251409455
82	ENSDARG00000092157	Uncharacterized protein	2.232082698
83	ENSDARG00000093437	SOBP	2.213737179
84	ENSDARG00000086554	Uncharacterized protein	2.197779134
85	ENSDARG00000093279	*si:dkey-11o1.3*	2.124256256
86	ENSDARG00000058256	*draxina*	2.122419282
87	ENSDARG00000063731	*rbm45*	2.098162736
88	ENSDARG00000035609	*mtnr1c*	2.080541925
89	ENSDARG00000042090	PLA2G4C	2.073758793
90	ENSDARG00000091561	*si:dkey-156k2.4*	2.069899449
91	ENSDARG00000018621	*slc6a19a.1*	2.057235368
92	ENSDARG00000096579	*si:dkey-9c18.3*	2.053035362
93	ENSDARG00000093979	*si:dkey-71b5.5*	2.047231975
94	ENSDARG00000068997	NLRP6	2.045946654
95	ENSDARG00000036105	*si:dkeyp-92c9.2*	2.018475243
96	ENSDARG00000003820	*nr1d2a*	2.013580419
97	ENSDARG00000043237	*pdzd3b*	2.000926404

Table S1. Fold change of differential up-regulated genes in *mct8*-/- 6 dpf larvae (RNA-seq data).

Table S2. Differential expression of down-regulated genes.

	Transcript ID	Transcript Name	Fold Change
1	ENSDARG00000093934	*si:ch211-281k12.1*	0.496502756
2	ENSDARG00000097803	*mtrfr*	0.496200953
3	ENSDARG00000058159	*ipo8*	0.492300254
4	ENSDARG00000090091	*si:ch211-238e22.2*	0.492261275
5	ENSDARG00000094343	*si:dkey-20i20.11*	0.491937492
6	ENSDARG00000070439	*pde6ha*	0.490977092
7	ENSDARG00000070272	*casp10*	0.487728418
8	ENSDARG00000070726	*cnga3a*	0.486740075
9	ENSDARG00000034095	*si:dkey-59l11.10*	0.485605799
10	ENSDARG00000038742	RBP1	0.485052263
11	ENSDARG00000090769	*si:dkey-51d8.6*	0.483468127
12	ENSDARG00000076997	*stxbp4*	0.481787898
13	ENSDARG00000089419	*dcp2*	0.481332677
14	ENSDARG00000030896	*foxq1a*	0.478430849
15	ENSDARG00000002748	*sema6d*	0.478185611
16	ENSDARG00000093972	*ftr79*	0.475992423
17	ENSDARG00000073836	*si:ch211-161m3.7*	0.475373003
18	ENSDARG00000045414	*elovl2*	0.474583692
19	ENSDARG00000086847	*irgq1*	0.472871971
20	ENSDARG00000092091	*zmp:0000001315*	0.467754689
21	ENSDARG00000071684	*rx1*	0.466913324
22	ENSDARG00000086458	*hdac10*	0.46665651
23	ENSDARG00000092620	Uncharacterized protein	0.465946984
24	ENSDARG00000043993	KCNIP2	0.46539661
25	ENSDARG00000096135	*si:dkey-6a5.10*	0.453462538
26	ENSDARG00000087442	*znf1022*	0.448996928
27	ENSDARG00000060545	TMC8	0.444537149
28	ENSDARG00000097211	*si:ch211-248g18.6*	0.443334707
29	ENSDARG00000094511	*ccl20b*	0.442846203
30	ENSDARG00000058711	Uncharacterized protein	0.44198325
31	ENSDARG00000091013	*si:dkey-84h14.2*	0.441829257

32	ENSDARG00000086904	*si:dkeyp-4c4.1*	0.44020822
33	ENSDARG00000089948	ENDOD1	0.439586385
34	ENSDARG00000059786	*sa39717*	0.428119875
35	ENSDARG00000097844	*si:ch1073-488c15.2p*	0.425212699
36	ENSDARG00000096217	*si:dkey-51d8.9*	0.423164908
37	ENSDARG00000028012	TPMT	0.421597422
38	ENSDARG00000077372	*tfr1b*	0.421483656
39	ENSDARG00000092002	DHPS	0.420750712
40	ENSDARG00000068830	*zgc:172139*	0.413786554
41	ENSDARG00000036304	*dnaaf3l*	0.411979901
42	ENSDARG00000028664	*ahsa1a*	0.411432819
43	ENSDARG00000089715	*si:cabz01054394.7*	0.411192578
44	ENSDARG00000076043	*si:dkeyp-73d8.9*	0.405698458
45	ENSDARG00000007582	*olfcb1*	0.403990246
46	ENSDARG00000095302	*si:dkey-263m6.1*	0.403331354
47	ENSDARG00000057644	*adam8b*	0.401963393
48	ENSDARG00000015236	*pycr3*	0.397012929
49	ENSDARG00000004647	*spice1*	0.393342122
50	ENSDARG00000088069	*ifit17*	0.388605161
51	ENSDARG00000076745	*zgc:193811*	0.386186914
52	ENSDARG00000013022	*si:ch211-59h6.1*	0.385680037
53	ENSDARG00000087518	*si:dkey-261p22.1*	0.385449761
54	ENSDARG00000077960	*si:ch211-186e20.7*	0.378376796
55	ENSDARG00000074193	*si:dkeyp-85d8.5*	0.377142671
56	ENSDARG00000093110	*fgf1b*	0.374780139
57	ENSDARG00000091187	UIMC1	0.373605656
58	ENSDARG00000026505	*si:ch211-285c6.3*	0.371775513
59	ENSDARG00000091744	Uncharacterized protein	0.370714521
60	ENSDARG00000096988	ZFLNCG06754	0.370664225
61	ENSDARG00000090461	*si:ch211-197f20.1*	0.370292224
62	ENSDARG00000097750	*si:dkey-181d19.6*	0.369603663
63	ENSDARG00000091269	*si:ch73-27e22.8*	0.364365844
64	ENSDARG00000093354	*si:ch211-57i17.2*	0.357648446
65	ENSDARG00000087855	*si:ch211-209n20.3*	0.355993102

66	ENSDARG00000059235	*zgc:113274*	0.355529544
67	ENSDARG00000096186	*si:dkey-247i3.5*	0.353318629
68	ENSDARG00000095251	*si:dkey-31n13.3*	0.351024313
69	ENSDARG00000006092	*rad21a*	0.35069071
70	ENSDARG00000093964	*si:ch211-250k18.6*	0.341443433
71	ENSDARG00000091235	Uncharacterized protein	0.341002104
72	ENSDARG00000086999	Uncharacterized protein	0.340787789
73	ENSDARG00000078940	*pcdh2g12*	0.340545014
74	ENSDARG00000090368	*dre-mir-132-3*	0.339202488
75	ENSDARG00000093105	*si:ch211-133l5.6*	0.337620627
76	ENSDARG00000045737	*guca1g*	0.336801086
77	ENSDARG00000079997	*si:zfos-223e1.2*	0.33615304
78	ENSDARG00000092992	*si:ch211-204a13.1*	0.33247664
79	ENSDARG00000058375	*syce2*	0.331928433
80	ENSDARG00000039265	*arhgap4a*	0.32932446
81	ENSDARG00000079272	*tmem14cb*	0.32904379
82	ENSDARG00000051711	Uncharacterized protein	0.32814392
83	ENSDARG00000074546	*si:ch211-213a13.11*	0.323296779
84	ENSDARG00000002981	*cyp2aa4*	0.319929967
85	ENSDARG00000068382	*pglyrp5*	0.319083636
86	ENSDARG00000070011	*hce2l2*	0.312249798
87	ENSDARG00000077068	si:ch211-11p18.6	0.311875718
88	ENSDARG00000088819	*si:dkey-4j21.2*	0.309221911
89	ENSDARG00000089941	Uncharacterized protein	0.305651463
90	ENSDARG00000095674	*tmem123*	0.304114876
91	ENSDARG00000037995	*gdf3*	0.294250546
92	ENSDARG00000071604	*si:ch211-156p11.1*	0.293685682
93	ENSDARG00000086153	*me2*	0.28908284
94	ENSDARG00000041550	*pimr138*	0.287869055
95	ENSDARG00000005221	*desmb*	0.28451713
96	ENSDARG00000043168	*cela1.5*	0.2809738
97	ENSDARG00000097930	*si:ch211-161c3.6*	0.272027115
98	ENSDARG00000043173	*cela1.3*	0.26972032

99	ENSDARG00000093296	*znf1106*	0.269661107
100	ENSDARG00000095414	Uncharacterized protein	0.267745083
101	ENSDARG00000035797	*si:dkey-17m8.2*	0.261420812
102	ENSDARG00000039491	*cfap61*	0.260986652
103	ENSDARG00000096140	*znf1120*	0.259420435
104	ENSDARG00000090403	*cyp2aa8*	0.251574523
105	ENSDARG00000087358	*si:dkeyp-109b10.4*	0.249485054
106	ENSDARG00000092139	*znf1102*	0.247666201
107	ENSDARG00000096398	*si:ch211-276a17.5*	0.243442628
108	ENSDARG00000086895	*si:dkey-238g7.2*	0.241728836
109	ENSDARG00000071103	*si:dkey-222p3.1*	0.240487805
110	ENSDARG00000070396	*serpinb1l2*	0.236371485
111	ENSDARG00000088245	*si:dkey-81i14.1*	0.233706149
112	ENSDARG00000096876	*si:dkey-61o18.2*	0.233595965
113	ENSDARG00000044862	*opn1lw1*	0.229621998
114	ENSDARG00000052465	*dusp10*	0.229094353
115	ENSDARG00000086835	*ostf1*	0.225645162
116	ENSDARG00000095293	*si:dkey-265e7.1*	0.223313139
117	ENSDARG00000043294	GUCA1A	0.222080051
118	ENSDARG00000088185	*eif2b1*	0.221320246
119	ENSDARG00000057769	*pimr55*	0.220429931
120	ENSDARG00000087157	*cant1b*	0.219855351
121	ENSDARG00000086612	*si:ch73-269m14.4*	0.214741606
122	ENSDARG00000016713	*dhrs13l1*	0.211074152
123	ENSDARG00000091969	*gbgt1l6*	0.210054273
124	ENSDARG00000086512	*prodhb*	0.207502673
125	ENSDARG00000042698	*si:dkey-15j16.6*	0.205954304
126	ENSDARG00000044629	*guca1d*	0.205070697
127	ENSDARG00000096094	*znf1105*	0.200264042
128	ENSDARG00000095253	Uncharacterized protein	0.199005753
129	ENSDARG00000095233	*si:dkeyp-51g9.4*	0.198983608
130	ENSDARG00000056502	*si:ch73-334d15.4*	0.197840914
131	ENSDARG00000091912	*si:ch211-15j1.5*	0.197472035
132	ENSDARG00000092889	*zgc:194246*	0.197326462

133	ENSDARG00000083100	*mir145*	0.192923004
134	ENSDARG00000062906	*kcnv2b*	0.192221497
135	ENSDARG00000087800	*si:dkeyp-82b4.2*	0.191028661
136	ENSDARG00000092239	*si:dkey-242k1.4*	0.190608023
137	ENSDARG00000075395	*dguok*	0.186444128
138	ENSDARG00000011701	*ctsll*	0.186380646
139	ENSDARG00000095949	*si:dkey-22i16.9*	0.185026612
140	ENSDARG00000044280	*opn1mw2*	0.181120694
141	ENSDARG00000077018	*socs7*	0.177392028
142	ENSDARG00000003523	*itln3*	0.174391639
143	ENSDARG00000071651	*si:dkey-19a16.13*	0.174360697
144	ENSDARG00000087315	Uncharacterized protein	0.169037767
145	ENSDARG00000092657	*si:ch211-276i6.1*	0.165305971
146	ENSDARG00000089625	C17orf67	0.160092099
147	ENSDARG00000056498	*crp*	0.159135608
148	ENSDARG00000096166	*si:dkey-29p23.2*	0.141630276
149	ENSDARG00000086984	Uncharacterized protein	0.139133379
150	ENSDARG00000076763	*sp2*	0.133195125
151	ENSDARG00000076252	*si:dkey-247i3.1*	0.13267883
152	ENSDARG00000097961	*si:dkey-86k10.6*	0.130234368
153	ENSDARG00000090743	Uncharacterized protein	0.130180605
154	ENSDARG00000087080	*cabz01044356.1*	0.129158852
155	ENSDARG00000095529	*gusb*	0.125236812
156	ENSDARG00000036895	*dap1b*	0.124470143
157	ENSDARG00000056462	*crp2*	0.119291425
158	ENSDARG00000079544	*si:ch1073-464p5.5*	0.118481343
159	ENSDARG00000052037	*trim35-1*	0.117875233
160	ENSDARG00000005959	*echdc1*	0.117412389
161	ENSDARG00000068841	*si:dkey-29p23.1*	0.117095781
162	ENSDARG00000002293	*si:ch211-197g15.9*	0.116376884
163	ENSDARG00000091000	*si:dkeyp-44b5.1*	0.113275435
164	ENSDARG00000095390	*si:dkey-264b2.2*	0.105362896
165	ENSDARG00000092811	*si:dkey-167i21.4*	0.103997606

166	ENSDARG00000078753	*sa7325*	0.101449929
167	ENSDARG00000091778	*si:dkey-86k10.4*	0.098485304
168	ENSDARG00000091811	*si:dkey-86k10.4*	0.098097236
169	ENSDARG00000096091	*si:dkey-29j8.3*	0.096899303
170	ENSDARG00000089304	*si:dkeyp-4f2.1*	0.091422454
171	ENSDARG00000088888	*znf994*	0.087660984
172	ENSDARG00000043540	*si:dkey-207l24.2*	0.087513344
173	ENSDARG00000094297	*si:dkey-222h21.5*	0.078308953
174	ENSDARG00000077415	*zmp:0000001175*	0.074039473
175	ENSDARG00000088673	Uncharacterized protein	0.073166577
176	ENSDARG00000095574	Uncharacterized protein	0.069018871
177	ENSDARG00000095088	LOC799039	0.064543669
178	ENSDARG00000086858	*pxna*	0.064123275
179	ENSDARG00000097997	*si:ch211-272b8.7*	0.064119709
180	ENSDARG00000090706	*slc1a5*	0.054728264
181	ENSDARG00000094001	*si:dkey-6a5.3*	0.053933915
182	ENSDARG00000071368	*si:dkey-86k10.3*	0.037811602
183	ENSDARG00000094747	*ifi44f5*	0.029668267
184	ENSDARG00000071894	*pcdh2g3*	0.026252683
185	ENSDARG00000097036	*si:dkey-86k10.5*	0.026195325
186	ENSDARG00000093361	Uncharacterized protein	0.025768057
187	ENSDARG00000071895	*pcdh2g1*	0.024982932
188	ENSDARG00000097976	*si:ch211-282f6.1*	0.01312704
189	ENSDARG00000070473	*parp6b*	0.012498864
190	ENSDARG00000087327	*cbx8a*	0.010420248
191	ENSDARG00000071629	Uncharacterized protein	0.007064597
192	ENSDARG00000088324	Uncharacterized protein	0.004353201
193	ENSDARG00000011652	*si:zfos-979f1.2*	0
194	ENSDARG00000091988	*si:dkey-164n2.1*	0
195	ENSDARG00000086998	*zgc:64002*	0
196	ENSDARG00000090975	*si:dkey-62k3.5*	0
197	ENSDARG00000096418	*ighv14-1*	0

198	ENSDARG00000089208	Uncharacterized protein	0
199	ENSDARG00000075260	Uncharacterized protein	0
200	ENSDARG00000055263	Uncharacterized protein	0
201	ENSDARG00000095179	*si:ch211-235f1.3*	0
202	ENSDARG00000096723	*si:ch73-91k6.7*	0
203	ENSDARG00000093446	*si:dkey-204f11.49p*	0
204	ENSDARG00000097770	*si:ch211-167j9.4*	0

Fold change of differential down-regulated genes in *mct8*-/- 6 dpf larvae (RNA-seq data).

Table S3. Significantly enriched gene pathways.

**Ingenuity Canonical Pathways**	**-log(*p-value*)**
1,25-dihydroxyvitamin D3 Biosynthesis	4.8
Necroptosis Signaling Pathway	2.24
Tetrahydrobiopterin Biosynthesis I	2.16
Tetrahydrobiopterin Biosynthesis II	2.16
Phototransduction Pathway	2.15
Retinoate Biosynthesis II	2.03
Proline Biosynthesis I	2.03
Endothelin-1 Signaling	2
Proline Biosynthesis II (from Arginine)	1.94
Arginine Degradation VI (Arginase 2 Pathway)	1.86
VDR/RXR Activation	1.85
Osteoarthritis Pathway	1.77
Death Receptor Signaling	1.68
Huntington's Disease Signaling	1.56
p38 MAPK Signaling	1.51
Sphingosine-1-phosphate Signaling	1.5
DNA Double-Strand Break Repair by Homologous Recombination	1.49
Pancreatic Adenocarcinoma Signaling	1.46
Adipogenesis pathway	1.41
Hereditary Breast Cancer Signaling	1.37
Endocannabinoid Cancer Inhibition Pathway	1.35
Inflammasome pathway	1.34
The Visual Cycle	1.3

Significantly enriched gene pathways in *mct8*-/- larvae using Ingenuity Pathway Analysis.
